# What are the barriers and facilitators for third sector organisations (non-profits) to evaluate their services? A systematic review

**DOI:** 10.1186/s13643-018-0681-1

**Published:** 2018-01-22

**Authors:** Anders Malthe Bach-Mortensen, Paul Montgomery

**Affiliations:** 1 Department of Social Policy and Intervention, University of Oxford, 32 Wellington Square, Oxford, OX1 2ER UK; 2 Department of Social Policy, Sociology and Criminology, University of Birmingham, B15 2TT, Birmingham, UK

**Keywords:** Evidence-based practice, EBP, Barriers and facilitators, Evaluation capacity, Third sector organisations, TSO, Non-profit, Evaluation practice, Third sector, NGO

## Abstract

**Background:**

The third sector is becoming a more common provider of social and health services, but little is known about how third sector organisations (TSOs) evaluate their activities. Past research has reported that the third sector is under increasing pressure to evaluate its impact and performance by government and other commissioning bodies. However, in responding to this increased pressure to undertake evaluation, research suggests that many TSOs struggle to evaluate their activities following the principles of evidence-based practice (EBP). Yet, there has been no systematic effort to investigate *why* the third sector is struggling to provide good quality evidence of its effects.

**Methods:**

This systematic review is reported following the PRISMA guidelines. Ten interdisciplinary databases were searched using a search string developed following best practice and in consultation with an information systems expert. Included studies were primary research of any research design investigating barriers to and facilitators of the evaluation process of TSOs as identified by practitioners. All studies were quality appraised, and the results were synthesised as a thematic summary.

**Results:**

Twenty-four studies were included, which mainly investigated TSOs working within health and social services. The thematic summary identified the main barriers for TSOs to undertake evaluation to be related to the (1) lack of financial resources, (2) lack of technical capability and evaluation literacy and (3) challenges around identifying relevant evaluation systems and outcome indicators. Key facilitating factors involved (1) getting the appropriate support, (2) having an organisational culture that supports evaluation and (3) the motivation to be accountable to stakeholders. These findings were robust to study quality.

**Conclusions:**

This review constitutes the first systematic effort to synthesise existing literature on factors supporting and preventing evaluation by TSOs. The prevalence of factors revolving around the lack of support, resources and clarity on appropriate outcome indicators suggests that many of the identified challenges may be met by applying evidence-based and stakeholder-inclusive strategies to develop shared evaluation requirements. Future efforts should address the application of EBP as part of the commissioning process of TSOs.

**Electronic supplementary material:**

The online version of this article (10.1186/s13643-018-0681-1) contains supplementary material, which is available to authorized users.

## Background

The third sector is under increasing pressure to report on its activities, partly in response to funding/commissioning requirements [1–8]. However, despite the increasing emphasis on impact and performance evaluation by government and commissioners, many third sector organisations (TSOs) fail to evaluate their activities following the principles of evidence-based practice (EBP), which promote rigorous, reproducible and systematic methods [9]. While it is generally acknowledged that monitoring and evaluation according to best practice is important in the delivery of services, there has been little systematic effort to investigate *why* the third sector is struggling to evidence its impact, despite becoming a growing provider of social and health services [10–13].

The ‘third sector’ is a vague and inconsistently applied term, which covers a range of different types of organisations, such as *voluntary organisations*, *community-based organisations*, *non-profits* and *charities*, which can each be argued to entail distinct nuances [14, 15].

In the context of this review, we will use the broader term ‘third sector organisation’ (TSO) and follow the structural/operational definition of TSOs [16], which can be summarised as “…organisations which are formally organised; non-profit distributing; constitutionally independent from the state; self-governing and benefiting from some form of voluntarism (e.g. with volunteer (unpaid) Trustees or Board members or using volunteers in the delivery of services)” [17].

It has been estimated that there are around 166,000 registered TSOs in the UK alone [18], but there is no commonly accepted estimate on the full size of the third sector worldwide. The third sector is becoming an increasingly common provider of public services [7, 19, 20], especially in social care [21, 22], and is considered a central actor in alleviating social issues in most Western countries [7, 23–26]. For example, the primary activities of many TSOs in the UK focus on the delivery of *social services* [18]. Further, the UK third sector constitutes a substantial workforce of around 853,000 employees (2016), with an annual spending of £43.3 billion (2014/2015) of which 21% (£9.7 billion) is spent on *social services* and 10.2% (£4.64 billion) on *health*. Of its annual income (£45.5 billion in 2014/2015), it has been estimated that the government provides £15.3 (33.6%) billion to the third sector, largely through contract-based commissioning. The main beneficiaries of the UK third sector activity are vulnerable groups such as *children and young people*, *the elderly* and *people with disabilities* [18].

Recent research and reports demonstrate that many TSOs fail to evidence their activities [27–29]. For example, a 2010 report developed by the Charity Finance Group (CFG) found that only 8% of their sample of 75 TSOs provided external evidence on their impact [28]. More recently, another report which surveyed 1000 TSOs found that 25% did not evaluate their work at all [27]. While these studies cannot be assumed to be representative of the full population of TSOs, they seem to mirror a growing body of evidence indicating that the sector struggles to adhere meaningfully to the increasing demand for the evaluation of their services [2, 13, 30, 31].

However, few efforts have addressed *why* TSOs that engage in evaluation struggle to demonstrate their impact to stakeholders despite the importance of understanding effectiveness (or possible harms) to vulnerable service users. To improve the current understanding of third sector practice, research is needed to explore the evaluation process and capacity of TSOs to shed light on what barriers and facilitators these organisations face. To date, there has been no systematic review aggregating the research on the barriers and facilitators for TSOs to undertake evaluation, which constitutes an important research gap since identifying these may be an important step in *improving* current practice and in assessing *how* TSOs may engage in better evaluation practice.

### What has been done?

Although there is currently little systematic evidence addressing evaluation practice (or lack thereof) by TSOs, a few studies have investigated the perceived barriers and facilitators of engaging in evaluation [27–29]. This existing research identified factors such as lack of financial resources and support, lack of technical expertise and poor availability of appropriate evaluation and impact tools [27, 28] as being central barriers for TSOs to undertake evaluation. Such barriers are often perceived to be grounded in the failure of funders and regulators to support TSOs in the evaluation process [29, 31]. However, no study has attempted to aggregate the full body of evidence on these factors.

Rather than focusing on the specific challenges of the *evaluation process* of TSOs, past research has focused on the barriers to and facilitators of the *use* and *mobilisation* of research and evidence by TSOs [17] and policy-makers [32, 33]. For example, Hardwick et al. [17] investigated the knowledge base in terms of how TSOs utilise research in their activities. This scoping review which considered neither study quality nor importance of the identified factors found a range of predominantly qualitative studies, which identified various perceived barriers to the use of evidence by TSOs that were mostly related to finance, expertise, resources and poor collaboration [17]. Perceived facilitators included knowledge mediation, practitioner involvement and intervention and implementation description. However, the focus of the review was on the knowledge mobilisation of third sector practitioners and not on the *evaluation process* [17]. While there might be important similarities to the barriers and facilitators associated with the use of evidence and the evaluation process of TSOs, these aspects are fundamentally different, in that TSOs might evaluate their activities without applying evidence as part of one’s practice, and vice versa. Further, focusing entirely on the use of evidence may overlook the issue of evaluation capacity.

### Objectives of the review

This systematic review addresses the following question: What barriers and facilitators do third sector practitioners identify in relation to evaluating the services their organisations provide?

## Methods

We conducted a systematic review in line with the PRISMA guidelines (see Additional file 1 for a completed checklist).

### Search strategy

High sensitivity was the main aim of the search which was designed to capture all existing research on the barriers and facilitators that TSOs experience in undertaking evaluation. Initially, we conducted preliminary reference list checks and hand-searched/browsed selected journals and databases to locate studies that an optimal search would ideally identify. Then, a range of third sector and methodology experts were contacted and invited to provide feedback on the protocol (available upon request) and search strategy (Additional file 2). This then informed the online search in a range of clinical and social science databases. The final search string was developed in consultation with an information expert. The included databases were the following:

- ABI/INFORM Global
- Applied Social Sciences Index and Abstracts (ASSIA)
- International Bibliography of the Social Sciences (IBSS)
- MEDLINE^®^
- PAIS Index
- Policy File Index
- Social Services Abstracts
- Worldwide Political Science Abstracts
- SCOPUS
- Open Grey

### Study selection

Included studies were primary research of any research design or systematic reviews that investigated barriers to and facilitators of the evaluation process by TSOs as identified by practitioners. In the context of this review, evaluation is defined as any attempt to quantify or evidence the impact, performance or outcomes of services provided by TSOs [1, 30, 34, 35]. As a result, this review will not be concerned with the distinct nuances between e.g. programme or outcome evaluation and performance measurement [36], effectively assuming that the barriers to different types of evaluation can be generalised. Thus, evaluation types such as performance management, evaluation capacity, impact assessment, outcome, programme or service evaluation were all considered for inclusion.

Barriers and facilitators are defined as any factors that prevent or support the evaluation capacity of third sector practitioners; they did not need to be the primary outcome of interest of a study to be considered for inclusion.

The third sector and TSOs are denoted inconsistently, and this study considered all synonyms of TSOs, such as *voluntary organisations*, *community-based organisations*, *non-profits* and *charity*. Studies that focus on barriers and facilitators in the *use* of evidence by TSOs were excluded, as the focus of this review is on the challenges and needs of TSOs to engage in evaluation. For practical reasons, studies needed to be available in English. There were no date restrictions for studies to be included in the review.

### Screening and data extraction

All abstracts were screened using the online tool Rayyan, and data were extracted by ABM with a 20% random sample screened independently by PM. Disagreements were resolved by consensus.

The following information was extracted from the included studies:

- Publication year and author
- Study aims
- Study design (time frame/data collection/data analysis)
- Population (type of organisations/services/sample size)
- Results (barriers/facilitators)
- Other results

### Data synthesis

The focus of this review is on factors identified by third sector practitioners, and included studies were thus either qualitative or survey-based or mixed methods. To synthesise this evidence, the review conducted a thematic summary (sometimes referred to as ‘narrative synthesis’), in view of the different types of research studies [37]. This included categorising the identified factors that support and prevent the evaluation capacity of TSOs into themes, in line with previous reviews of this type [32, 38, 39]. This process allowed for a more thorough utilisation of the extracted data, as many of the identified factors could potentially circulate around the same underlying problems, which might be overlooked if the significance of identified factors were only counted by individual frequency. To test the robustness of the thematic framework and to provide an overview of how the individual studies contributed to the construction of the identified themes, we also constructed a table following Rees et al. [40]. This enabled an assessment of whether certain studies were over- or underrepresented in the thematic framework and helped identify whether methodology was related to certain themes [37].

### Quality assessment

To assess the quality of cross-sectional studies, the review employed the recently developed AXIS checklist [41] (see Appendix A in Additional file 3). For the appraisal of interview, ethnography and focus group studies, an adapted version of the CASP checklist for qualitative studies and the Joanna Briggs Institute QARI checklist was employed (Appendix B in Additional file 3) [42]. It is important to note that quality appraisal will always entail a degree of value judgement, and that the comparison of the quality of different types of studies (e.g. qualitative and quantitative) may be somewhat arbitrary. Yet, the assessment of study quality arguably allows for a more robust assessment of the findings and of whether the identified factors vary across studies of different quality [37]. Ultimately, appraised studies were organised into three categories—high, medium and low. The basis of the quality ranking was informed by the number of checklist items fulfilled by the individual studies, but the final assessment of quality was based on an overall judgement of the value of the individual findings, considering the methodology of the included studies.

## Results

**Fig. 1:**
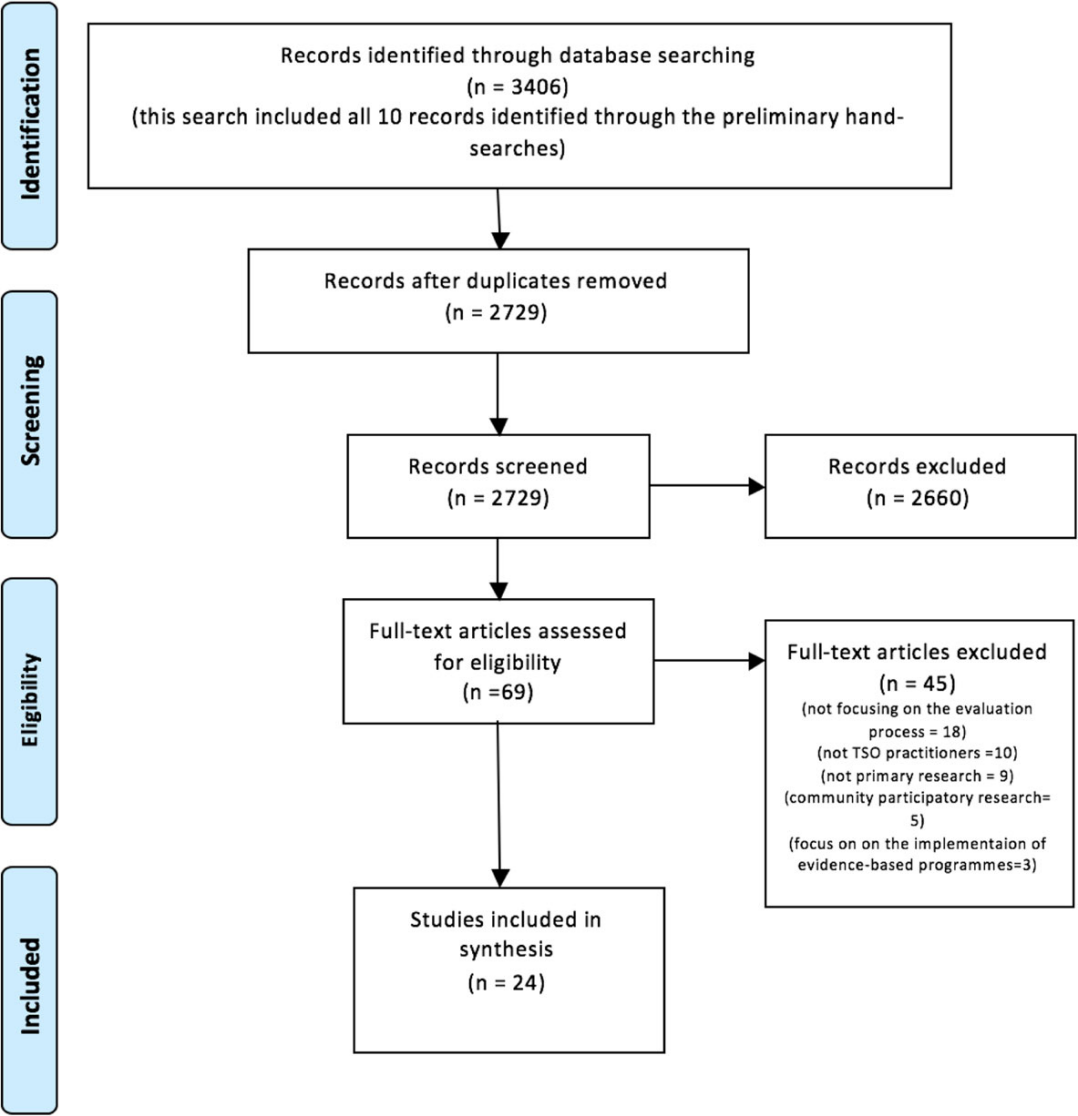
PRISMA diagram

Prior to the formal search, ten potentially eligible studies had been identified through preliminary scoping of reference lists and hand searches. The final search located 3406 studies of which 2729 unique studies were identified after removing duplicates. Of these, 2660 were excluded after screening the abstracts. Sixty-nine studies were then read in full text of which 45 were excluded because of the following reasons: not focusing on the evaluation process of TSOs (*n* = 18), not representing the experiences of TSO practitioners (*n* = 10), not being primary research (*n* = 9), focusing on the collaboration between researchers and community programmes (*n* = 5) or focusing on the implementation of evidence-based procedures rather than evaluation of services (*n* = 3). Twenty-four studies were included in the final synthesis (Fig. 1).

### Characteristics of included studies

The full characteristics and extracted data of the included studies can be found in Additional file 4. Of the 24 included studies, nine were reports written by and/or for third sector organisations and 15 were articles published in peer-reviewed journals. Nine studies (37.5%) were purely quantitative, nine were purely qualitative (37.5%) and six employed mixed methods (25%). The clear majority of studies (70.8%) were conducted in North America, followed by the UK (20.8%). Only one study (4.2%) was conducted entirely in a low and middle-income country (LMIC), and one study (4.2%) was conducted in multiple contexts—USA, Columbia, Guam and Puerto Rico. Eight of the studies (33.33%) were published in the period between 2000 and 2009 and 16 studies (66.7%) after 2010.

### Samples of TSOs in included studies

**Fig. 2:**
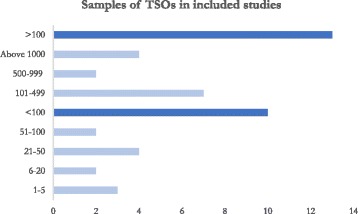
Samples of TSOs in included studies

Thirteen of the included studies (54.2%) had a sample size above 100 TSOs, and only five (20.8%) included less than 20 TSOs. Of the studies with a sample < 100, three had a sample of 1–5 TSOs, two contained 6–20 [43, 44], four a sample of 21–50 [45–48] and two a sample of 51–99 [49, 50]. Of the studies with a sample > 100 TSOs, seven included 100–499 TSOs [28, 51–55], two included 500–999 TSOs [29, 56] and four a sample of 1000 or more TSOs [27, 57–59] (Fig. 2).

### Size of TSOs

Seventeen of the studies (70.8%) included a mixed population of TSOs in terms of income [27–29, 43, 44, 46, 47, 49, 51–53, 55–59], of which eight had a self-reported bias towards larger organisations [27, 28, 44, 47, 56, 58, 59]. Two studies [60] only included small TSOs (annual income below £100,000), one study [48] focused on small- to medium-sized TSOs (£100,000–£1 million) and one report [61] looked at medium to large organisations (above £1 million). Three studies [45, 50, 54] did not specify the income of their included organisations, as their focus was on the evaluation capacity of organisations in the context of the implementation of specific programmes (e.g. ‘Healthy Tomorrow’ [54]).

### Purpose of TSOs

**Fig. 3:**
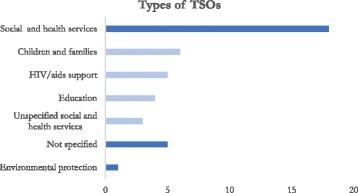
Type of service delivery of the organisations included in the studies

Most of the included studies (70.8%) investigated TSOs working primarily in social and human services (e.g. health, children and families, HIV/AIDS), followed by six studies (25%) that did not specify the included population of TSOs and one study (4.2%) working with environmental preservation organisations. Seven of them (29.2%) included mixed types of organisations in their analysis, of which organisations delivering social services constituted the biggest proportion. The biggest reported sub-categories of social services were organisations working with children and families (25%), HIV/AIDS service organisations (20.8%) and education (16.7%) (Fig. 3).

### Type of evaluation

All studies investigated evaluation in some way but used different terminologies on the specific *type* of evaluation. Six studies (25%) investigated *performance evaluation*, five studies (20.8%) looked at *evaluation capacity*, eight studies (33.3%) focused on *evaluation practice*, four studies (16.7%) on *programme evaluation* and one study (4.2%) focused on *impact evaluation*.

### Quality of included studies

All 24 studies were subject to quality appraisal. Three studies (12.5%) were deemed to be of *high quality* [31, 50, 61] and 15 (62.5%) of *medium quality* [27, 29, 43–47, 49, 51, 54, 55, 57–59], and the remaining six studies (25%) [28, 48, 53, 56, 60] were assessed to be of *low quality*. Of the studies that employed mixed methods, the appraisal focused on the methodology which identified the factors operating as barriers and facilitators. Most of the mixed methods studies were predominantly surveys [28, 29, 31, 53, 55, 57] but included interviews and/or focus groups to inform or validate the findings and were consequently appraised using cross-sectional criteria.

#### Identified barriers and facilitators

All 24 studies investigated factors operating as barriers for TSOs to evaluate their activities, whereas only 15 of those studies included factors that facilitate evaluation among TSOs. Only two studies [44, 50] identified the factors operating as barriers and facilitators as being reciprocal, whereas all other studies found them to be distinct. The synthesis identified 30 unique factors operating as barriers and 26 factors operating as facilitators. Interestingly, many of the identified barriers and facilitators did not mirror each other, as is often the case in reviews of this type [32, 39].

The top three cited factors operating as barriers to engagement in evaluation focused on the lack of expertise and internal capacity (17/24), mismatch between funder requirements and what TSOs perceived to be appropriate evaluation goals (16/24) and the lack of financial resources to conduct evaluation (14/24). The most reported factors operating as facilitators to evaluation included funders requiring evaluation (6/15), involving stakeholders in identifying relevant outcome indicators and evaluation goals (5/15) and having appropriately trained staff to undertake evaluation (5/15) (Table 1).

**Table 1:** Top three cited barriers and facilitators

Top 3 reported barriers
Lack of expertise and internal capability (17/24)
Mismatch between funder requirements and appropriate goals (16/24)
Lack of financial resources (14/24)
Top 3 reported facilitators
Funder requirements (6/15)
Involvement of stakeholders to identify outcome indicators and evaluation goals (5/15)
Training of staff and evaluation literacy (5/15)

### Weight of factors

While most of the studies did not rank the identified factors hierarchically, some studies did argue that certain factors were more significant than others, which was based on that factor being more frequently reported by respondents. Of the studies that did rank identified barriers, *lack of time* was the factor that most studies argued to be the *main* barrier to engage in evaluation (*n* = 6) [29, 30, 52, 56, 58, 59]. For facilitators, the factor most often reported as the *main* facilitator to undertake evaluation was having an organisational management that supports and requires evaluation (*n* = 2) [57, 58].

#### Themes

Many of the reported barriers and facilitators were described differently but revolved around the same underlying problem (e.g. the lack of financial and staff resources may reflect similar constructs). To approach the fact that many factors revolve around similar fundamental issues, we organised all the identified factors into overarching categories (Table 2), in line with the purpose of this type of review [32, 38, 39].

**Table 2:** Factors operating as barriers and facilitators across identified themes

	Times cited
Factors operating as barriers	
Factors related to lack of resources	36
Financial resources	14
Staff resources	6
Lack of time	6
Lack of resources to hire external evaluators	3
Resources (unspecified)	6
Staff turnover	1
Factors related to technical capability and evaluation skills	36
Lack of expertise and internal capability	17
Problems with data collection and analysis	11
Inability to utilise existing data	3
Difficulty conceptualising and designing evaluation	1
Technical challenges	4
Challenges in utilising evaluation systems and identifying outcome indicators	27
Difficulty developing and using evaluation tools	5
Lack of integrated systems to collect and analyse data	9
Challenges in identifying accepted outcome and impact indicators	13
Factors related to organisational culture and management	26
No perceived benefit to conduct evaluation	1
Staff resistance to evaluation	10
Perceived compromise between evaluation and service delivery	3
Lack of evaluation strategies and planning	3
Lack of feedback between board and management staff	1
Low prioritisation of evaluation	3
Lack of support from board and leadership	4
Evaluation not part of everyday practice	1
Factors related to funder requirements	25
Lack of funder requirements and support	3
Differing requirements from different funders	1
Mismatch between funder requirements and appropriate goals	16
Poor proportioning of size of charities and funder requirements	1
Micro-management by donors	1
Funding insecurity (funding circles incentivising focusing on immediate outputs rather than long-term outcomes)	3
Other	5
Confidentiality of data	3
Lack of cooperation with stakeholders	2
Factors operating as facilitators	
Factors related to receiving support to evaluate	19
Partnering with evaluation experts	3
Partnering with organisations working with similar activities	1
Technology availability and literacy to collect and analyse data	4
Benchmark data availability	2
Training of staff and evaluation literacy	5
Workable evaluation tools	2
Having sufficient resources to evaluate	2
Factors related to organisational culture and management	18
Understanding internal processes	3
To embed evaluation as part of everyday practice	1
Improve allocation of resources	1
Support from board and leadership	4
Have in-house evaluation staff	3
Having clear goals and evaluation strategies	2
Staff support	3
Positive perception of evaluation	1
Factors related to the motivation to be accountable	17
Involvement of stakeholders to identify outcome indicators and evaluation goals	5
The motivation to influence policy	1
Compare work and outcomes to others doing similar work	1
The motivation to inform the sector as whole	1
Improve targeting of beneficiaries	1
Identify new approaches	1
Ensuring control and legitimacy of activities to stakeholders	3
The motivation to demonstrate and improve effectiveness of services	4
Factors around funder requirements and regulations	7
Funder requirements	6
Regulation requirements	1
Factors around economic sustainability	5
Using evaluation to be eligible for funding opportunities	3
Using evaluation as marketing	2

As Table 2 shows, the two biggest categories preventing TSOs from undertaking evaluation were factors associated with the lack of resources (e.g. lack of money, time and staff) (*n* = 36) and the lack of technical capability and skills to evaluate (*n* = 36). The third biggest theme was challenges related to utilising evaluation systems and identifying relevant impact and outcome indicators (*n* = 27). Factors linked to organisational culture was also a significant theme (*n* = 26), which included staff resistance to evaluation and lack of support to engage in evaluation from the board and leadership. Another significant theme included factors associated with funder requirements (*n* = 25), which was mainly constituted by the perceived inappropriateness of the evaluation and reporting requirements set by commissioners.

The most reported category of facilitating factors focused on getting the appropriate support to undertake evaluation (*n* = 19), which included receiving expert support in evaluating services and receiving staff training to ensure the appropriate literacy to run evaluations. The second most reported category was factors related to organisational culture and management, which included staff and management support to undertake evaluation. The third most salient category of facilitating factors was motivation to be accountable (*n* = 17), which mainly revolved around including stakeholders in defining outcome and impact criteria, as well as ensuring control and effectiveness of services. Lesser reported factors facilitating evaluation were those related to the requirements set by funders and regulators (*n* = 7) and economic sustainability (*n* = 5).

### Robustness of themes

Tables 3 and 4 illustrate that the salience of themes corresponded to how representative the themes were of the included studies. However, *organisational culture and management* was an exception, in that it represented more of the included studies, compared to other top categories of facilitators. Notably, it was only survey studies which did not recognise *challenges around identifying appropriate outcome indicators* as a barrier [49, 53, 56, 58, 59], and only two studies, both qualitative, did not identify *resources* as a barrier to evaluation [43, 61]. However, the tables do not reflect any clear pattern to suggest that methodology or sample size systematically affects how the included studies contribute to the specific themes.

**Table 3:**
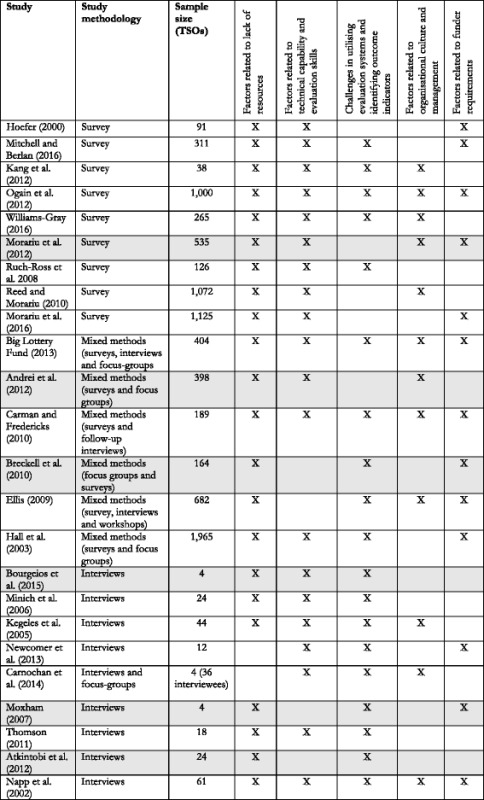
Overview of the contribution of individual studies on the identified themes of barriers

The table reflects how each study contributed to the construction of the final themes of factors operating as barriers. However, it should be noted that each study can contribute with multiple factors to the same theme. Shading indicates studies that were rated to be of low quality

**Table 4:**
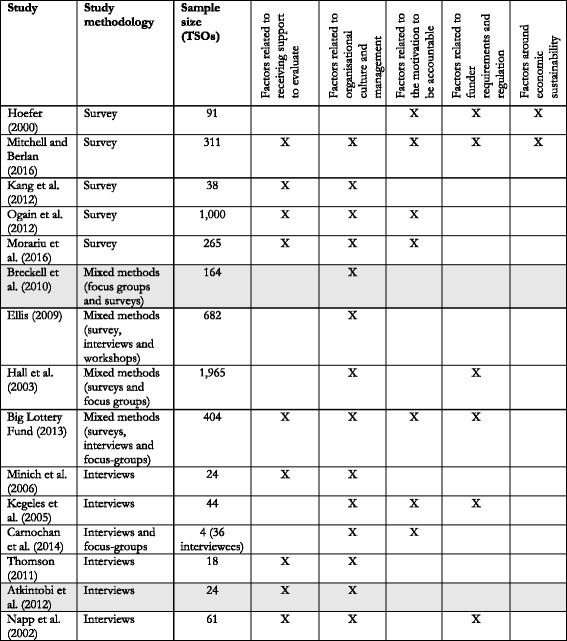
Overview of the contribution of individual studies on the identified themes of facilitators

The table reflects how each study contributed to the construction of the final themes of factors operating as facilitators. However, it should be noted that each study can contribute with multiple factors to the same theme. Shading indicates studies that were rated to be of low quality

### Sensitivity analysis

To test for the sensitivity of the results according to study quality, a sensitivity analysis was conducted by including only the *high*- or *medium*-quality studies. The top three main reported factors operating as facilitators and barriers remained the same (only two of the studies of low quality included facilitating factors) (Appendix C in Additional file 3). Looking at how excluding the studies of low quality affects the thematic categories (Appendix D in Additional file 3), the most reported themes still revolved around technical capability and evaluation skills (*n* = 28), factors associated with resources (*n* = 26) and factors associated with evaluation systems and outcome indicators (*n* = 21). The main categories of facilitating factors similarly remained the same after removal of low-quality studies, suggesting that the main identified categories are robust to this analysis.

## Discussion

### Summary of findings

This systematic review constitutes the first systematic attempt to synthesise existing research on what factors support or prevent evaluation for TSOs. The search identified 15 peer-reviewed publications and nine reports that can be classified as grey literature. The review identified the main factors preventing TSOs to undertake evaluation to revolve around the lack of financial resources, technical capability and evaluation literacy and challenges around identifying relevant evaluation systems and outcome indicators. The main facilitators involved getting appropriate support to evaluate, having an organisational culture and management in favour of evaluation and the pursuit of accountability. These findings were robust across different levels of study quality and were largely representative of the full body of included studies.

### Implications of findings

The thematic synthesis demonstrated that the main barriers for TSOs to undertake evaluation were related to organisational *capacity* and *capability*. One may think of capacity as *how much* an organisation can do, relating to issues around resources, and capability as *how well* an organisation can operate, thus relating to the skills, knowledge and confidence of organisations [62, 63]. These issues were reflected in the most reported categories of factors operating as barriers: *factors related to lack of resources* (capacity) and *factors related to technical capability and evaluation skills* (capability).

Importantly, these findings suggest that many TSOs are not receiving the appropriate capacity *or* capability support in evaluating their services. The significance of these barriers is further illustrated by the most reported categories of facilitating factors being to receive the appropriate support to undertake evaluation. The prevalence of these categories implies a rather unfortunate situation in which TSOs are faced with a growing pressure to evidence their performance to secure grants or contracts, but without being subject to the necessary support to undertake meaningful evaluation.

This situation might shed light on the increasingly reported tendency that many TSOs customise their evaluation procedures not to improve their service delivery but rather to satisfy funding bodies and to meet the increased pressure of performance measurement [27, 29, 31, 64, 65]. Having an incentive structure which encourages TSOs to shape evaluation entirely according to funder requirements is greatly problematic, as it may override what is arguably the most important pursuit of evaluation, i.e. to *monitor* and *improve* practice [66, 67]. Further, if those evaluation criteria are flawed or inappropriate to the activities to which they apply, the commissioning process may facilitate a market that incentivises poor and quick evaluations.

However, it was not only *external* barriers and facilitators that were identified as being central for TSOs to undertake evaluation. The themes of organisational culture and management were consistent across factors operating as both barriers and facilitators, particularly regarding staff resistance/support and organisational motivation (or lack thereof) to undertake evaluation. This finding is, for example, mirrored in a recent report by the Big Lottery Fund, which found that despite allowing its grantees to spend up to 10% of the allocated funding on evaluation, half of the surveyed TSOs (*n* = 404) only allocated between 1 and 5% of their budget to evaluation activities [55]. Improvement of evaluation capacity and capability among TSOs thus not only requires increased external support in terms of funders allowing for higher evaluation spending. This point is further exhibited by one of the most cited categories of facilitating factors being *organisational culture and management* (*n* = 18) and *the motivation to be accountable* (*n* = 17), suggesting that it is essential for TSOs to demonstrate *internal* motivation and will to mobilise existing resources towards improving evaluation capacity and capability [46, 50, 61].

This review also demonstrated that TSOs face considerable challenges regarding the unclear terminology and fluctuating understanding of terms such as ‘evaluation’ and ‘performance management’. This challenge is reflected by the second most reported barrier being the perceived mismatch between the requirements set by funders and what practitioners deemed to be valuable pursuits (16/24) and by one of the most reported facilitators being to involve stakeholders in the process of identifying outcome and impact indicators (5/15). This implies that when it comes to choosing *how to evaluate* and *what to measure*, there is poor consensus among funders, practitioners and other stakeholders about what constitutes *good practice*. Such perceived lack of consensus points to an absence of stakeholder collaboration and inclusion in defining and planning the evaluation requirement of third sector services.

#### Ways forward

The most cited groups of factors facilitating TSOs to undertake evaluation were getting the appropriate support to evaluate (*n* = 19), in which *financial support* was only identified twice as a facilitating factor under this theme. The low reporting of financial resources as a facilitating factor is noteworthy, considering that *lack of resources* (*n* = 36) was among the most cited categories of factors operating as a barrier, along with the lack of evaluation skills and capability (*n* = 36). This suggests that increasing financial support for TSOs to evaluate might be a necessary factor to improve evaluation practice, but that it is not sufficient in isolation. Rather, the analysis demonstrates that TSOs need improved support in the entire *evaluation process*, which would require attention to both capacity (more resources) and capability (e.g. staff training and collaboration with experts and researchers).

Further, there seems to be a need for clearer and more commonly accepted outcome indicators and evaluation guidelines that can be effectively utilised by third sector practitioners. Currently, there is a wide range of available evaluation frameworks and impact assessment tools made for and by TSOs [4, 68–73]. These tools include social returns on investment (SROI), social auditing and other types of outcome measurement [1, 70, 71, 74]. However, there is limited reliable data on the *use* and *uptake* of the various existing impact evaluation frameworks, and there are not any universal guidelines and consensus as to how different TSOs should apply them [1, 4, 26, 27, 29, 70, 74]. The findings of this review suggest that the failure to provide clear and consensus-based guidelines on the *appropriateness* of different types of evaluation procedures operate as a central barrier for TSOs to undertake evaluation.

The role of consensus and stakeholder inclusion is an essential aspect in adhering to the principles of EBP [75–77]. For example, when deciding on the appropriateness of new reporting and evaluation guidelines in clinical practice, methods such as the Delphi technique are widely considered to be best practice and served as the main tool to develop the CONSORT guidelines and its extensions [78–80]. While third sector scholars have continuously argued against the notion of a universal performance measurement, this review supports the idea that more efforts are needed to determine consensus-based and manageable criteria for outcome indicators and effectiveness in the third sector [8, 81]. Importantly, this does not necessarily require the adherence to strict or *one-size-fits-all* outcome or performance criteria. Rather, one might utilise consensus-based techniques such as the Delphi method to develop stakeholder-inclusive *processes*, from which an evaluation strategy can be defined by both service users, practitioners and funders. Further, engaging with stakeholders would constitute a central step in deciding what types of organisational support are necessary to ensure that TSOs can adhere to appropriate evaluation criteria. However, there is currently little research that addresses how consensus procedures from EBP can be utilised in the third sector.

### Limitations of this study

The main purpose of this study was to aggregate all available research addressing the current evaluation practice of TSOs and thus serves as an inclusive exploration of existing research investigating factors affecting third sector evaluation capacity and capability. Like most reviews of this type, the study depends on a form of *vote-counting*, in that the findings are constituted by the aggregation of factors reported as barriers and facilitators. However, by organising the factors into identified themes, the reported factors should be less sensitive to the, arguably, arbitrary difference between evaluation expertise and experiencing challenges with collecting and analysing data. Further, the three most reported barriers as well as the ranking of the themes remained consistent when removing the *low-quality* studies, which points to the overall robustness of the analysis.

It is important to note that many of the surveys suffered from either very low response rates [30, 58, 59], non-reporting of the response rates [28, 29, 53, 56] or the tendency to have a bias towards including larger organisations [27, 28, 44, 47, 56, 58, 59]. Also, in the cross-sectional studies, TSOs were restricted to the factors that were included (or not included) in the survey questionnaires. This constitutes a considerable limitation, as those factors might not have been the same if following an inductive or bottom-up qualitative approach. However, many of the cross-sectional studies included focus groups and interviews to ensure that the questions were reflective of issues that were recognised by TSO practitioners.

A more substantial limitation of this review is that different types of organisations might be subject to different factors preventing or supporting their evaluation capacity, which compromises the generalisability of the findings. For example, one of the included studies found a separate set of barriers between NGOs operating in Egypt and in Columbia [43]. Also, several studies found that smaller TSOs experience greater challenges in evaluating their services [27, 46, 52, 53]. To explore this tendency, one study [52] ran a logistic regression of different organisational characteristics to look for correlations in what factors were reported. This analysis found six organisational characteristics to be significantly associated with the inclination to report different types of barriers. However, when exploring these characteristics through cluster analysis, the study found that the size of organisations, type of services and the funding sources did not explain any variability between the reported barriers of the different clusters [52].

The included population of this review was predominantly TSOs from Western countries delivering social and health services. However, given that third sector spending tends to be higher in Western TSOs, these findings may be generalisable to a larger population of organisations [82, 83]. Moreover, the delivery of social services often includes the potential to cause harm to the service user [11, 12, 84, 85], and recent research suggests that TSOs delivering social services are more likely to be publically funded [86], thus making the evaluation capacity of this type of TSOs particularly important to investigate.

### Future research

This review focused on the perspectives of third sector practitioners, following the assumption that they would provide the most reliable accounts regarding factors influencing the evaluation capacity of TSOs. Future research might expand this focus to a wider group of stakeholders, which might shed light on how commissioning decisions are made in practice [87, 88]. Also, one might argue that the focus on factors operating as barriers and facilitators is restrictive, and future research might benefit from taking a broader approach by focusing on the overall *experiences* of TSOs to undertake evaluation. Last, this review did not investigate factors affecting the capacity for TSOs to *implement* evidence-based programmes and interventions, which is often acknowledged to be a central limitation in the third sector delivery of social and clinical work [13, 89]. Building on this review, future efforts should be made to investigate the barriers and facilitators related to the *implementation* of evidence-based interventions and programmes by TSOs.

## Conclusions

The main implication identified in this systematic review is the apparent willingness of funders and regulators to enforce evaluation on TSOs without offering support and consistent guidelines as to how evaluation should be undertaken. To address this problem, it is central not to focus on individual barriers (such as financial resources) but to consider what support is necessary to ensure that TSOs have the appropriate capacity and capability to undertake evaluation [62].

This may be achieved by having a procedure for consensus to determine, first of all, what evaluation criteria different types of TSOs require and, equally important, what types of supports will enable TSOs to conduct meaningful evaluation. Without the assurance that evaluation requirements are based on *best practice* and stakeholder perspectives, the utility of evaluation (i.e. to improve practice and prevent iatrogenic effects) is jeopardised, and the service users of the third sector are put at risk. To improve the current evaluation practice of the third sector, guideline development procedures employed by EBP may enable improved and consensus-based guidance that incorporates stakeholder perspectives with the best available research [80].

Most research on the performance of TSOs concludes that there is insufficient knowledge about current activities to make general inferences about the effectiveness of the third sector delivery of services [7, 17, 21]. This means that it is difficult to assess with confidence whether the increased third sector delivery of public and social services is indeed effective and worthwhile [7]. This paper is the first systematic review to investigate what factors obstruct and promote evaluation by TSOs, and as such, represents an important step in trying to *improve* current practice. The findings of this review may inform future efforts to investigate how current funding requirements can be adapted to facilitate the necessary evaluation capacity and capability for TSOs to evidence activities according to best practice.

## Additional files

Additional file 1: contains a completed PRISMA checklist. (DOC 64 kb)

Additional file 2: contains the search strategy employed by the study. (DOCX 98 kb)

Additional file 3: contains the appendices referred to throughout the study. (DOCX 99 kb)

Additional file 4: contains the extracted data that is analysed throughout the study. (XLSX 45 kb)

## Abbreviations

EBP: Evidence-based practice
TSO: Third sector organisation
