# Supplementary material for: What are the barriers and facilitators for third sector organisations (non-profits) to evaluate their services? A systematic review
Source: Syst Rev. 2018 Jan 22;7:13. doi: 10.1186/s13643-018-0681-1 (PMC5778760; doi:10.1186/s13643-018-0681-1)
Supplement: Supplementary file 2 — contains the search strategy employed by the study. (DOCX 98 kb) [file 13643_2018_681_MOESM2_ESM.docx]

## Search strategy for included databases

## Search string using ProQuest

| **Set#** | **Searched for** | **Databases** | **Results** |  |
| --- | --- | --- | --- | --- |
| S1 | TI(charity OR charities OR “charitable organisation*” OR “voluntary sector” OR “voluntary organisations” OR “third sector” OR third-sector OR “community organisation*” OR community-based OR “community based” OR non-profit* OR “not for profit” OR “nonprofit” OR non-profit “social enterprise*” OR TSO* OR CBO* OR VCO* OR VCS* OR "civic sector" OR "civil sector" OR "social sector" OR CSO* OR “Non Government*” OR NGO* OR NPO* OR “civil society”) | ABI/INFORM Global, Applied Social Sciences Index & Abstracts (ASSIA), International Bibliography of the Social Sciences (IBSS), MEDLINE®, PAIS Index, Policy File Index, Social Services Abstracts, Worldwide Political Science Abstracts | 75454° | |
| S2 | MESH(charity OR charities OR “charitable organisation*” OR “voluntary sector” OR “voluntary organisations” OR “third sector” OR third-sector OR “community organisation*” OR community-based OR “community based” OR non-profit* OR “not for profit” OR “nonprofit” OR non-profit “social enterprise*” OR TSO* OR CBO* OR VCO* OR VCS* OR "civic sector" OR "civil sector" OR "social sector" OR CSO* OR “Non Government*” OR NGO* OR NPO* OR “civil society”) | ABI/INFORM Global, Applied Social Sciences Index & Abstracts (ASSIA), International Bibliography of the Social Sciences (IBSS), MEDLINE®, PAIS Index, Policy File Index, Social Services Abstracts, Worldwide Political Science Abstracts | 9888° | |
| S3 | ALL((barrier* OR facilitat* OR challenge* OR benefit* OR success* OR constrain* OR difficult* OR enhanc* OR influen* OR interfer* OR motivat* OR obstruct* OR problem* OR promot* OR restrain* OR restrict* OR disincentive* OR factor* OR capacity) NEAR/4 (evaluate OR evaluation OR evaluating OR assess* OR impact OR measurement OR measuring OR experience* OR learning OR performance OR “EBP” OR “evidence based practice” OR “evidence-based practice” OR outcome)) | ABI/INFORM Global, Applied Social Sciences Index & Abstracts (ASSIA), International Bibliography of the Social Sciences (IBSS), MEDLINE®, PAIS Index, Policy File Index, Social Services Abstracts, Worldwide Political Science Abstracts | 848811° | |
| S4 | ALL((barrier* OR facilitat* OR challenge* OR benefit* OR success* OR constrain* OR difficult* OR enhanc* OR influen* OR interfer* OR motivat* OR obstruct* OR problem* OR promot* OR restrain* OR restrict* OR disincentive* OR factor* OR capacity) NEAR/4 (evaluate OR evaluation OR evaluating OR assess* OR impact OR measurement OR measuring OR experience* OR learning OR performance OR “EBP” OR “evidence based practice” OR “evidence-based practice” OR outcome)) AND (MESH(charity OR charities OR “charitable organisation*” OR “voluntary sector” OR “voluntary organisations” OR “third sector” OR third-sector OR “community organisation*” OR community-based OR “community based” OR non-profit* OR “not for profit” OR “nonprofit” OR non-profit “social enterprise*” OR TSO* OR CBO* OR VCO* OR VCS* OR "civic sector" OR "civil sector" OR "social sector" OR CSO* OR “Non Government*” OR NGO* OR NPO* OR “civil society”) OR TI(charity OR charities OR “charitable organisation*” OR “voluntary sector” OR “voluntary organisations” OR “third sector” OR third-sector OR “community organisation*” OR community-based OR “community based” OR non-profit* OR “not for profit” OR “nonprofit” OR non-profit “social enterprise*” OR TSO* OR CBO* OR VCO* OR VCS* OR "civic sector" OR "civil sector" OR "social sector" OR CSO* OR “Non Government*” OR NGO* OR NPO* OR “civil society”)) | ABI/INFORM Global, Applied Social Sciences Index & Abstracts (ASSIA), International Bibliography of the Social Sciences (IBSS), MEDLINE®, PAIS Index, Policy File Index, Social Services Abstracts, Worldwide Political Science Abstracts | 3372° | |

**Search string for SCOPUS**

Search 1:
TITLE-ABS-KEY ( charity  OR  charities  OR  "charitable organisation*"  OR  "voluntary sector"  OR  "voluntary organisations"  OR  "third sector"  OR  third-sector  OR  "community organisation*"  OR  community-based  OR  "community based"  OR  non-profit*  OR  "not for profit"  OR  "nonprofit"  OR  non-profit  "social enterprise*"  OR  tso*  OR  cbo*  OR  vco*  OR  vcs*  OR  "civic sector"  OR  "civil sector"  OR  "social sector"  OR  cso*  OR  "Non Government*"  OR  ngo*  OR  npo*  OR  "civil society" )

Hits: 4713

Search 2:
TITLE-ABS-KEY ( ( barrier*  OR  facilitat*  OR  challenge*  OR  benefit*  OR  success*  OR  constrain*  OR  difficult*  OR  enhanc*  OR  influen*  OR  interfer*  OR  motivat*  OR  obstruct*  OR  problem*  OR  promot*  OR  restrain*  OR  restrict*  OR  disincentive* )  AND  ( evaluate  OR  evaluation  OR  evaluating  OR  assess*  OR  impact  OR  measurement  OR  measuring  OR  experience*  OR  learning  OR  performance  OR  "EBP"  OR  "evidence based practice"  OR  "evidence-based practice"  OR  outcome ) )

Hits: 7,447,900

Search 3:
1+2

Hits: 1695

Search 4:
( TITLE-ABS- KEY ( ( barrier*  OR  facilitat*  OR  challenge*  OR  benefit*  OR  success*  OR  constrain*  OR  difficult*  OR  enhanc*  OR  influen*  OR  interfer*  OR  motivat*  OR  obstruct*  OR  problem*  OR  promot*  OR  restrain*  OR  restrict*  OR  disincentive* )  AND  ( evaluate  OR  evaluation  OR  evaluating  OR  assess*  OR  impact  OR  measurement  OR  measuring  OR  experience*  OR  learning  OR  performance  OR  "EBP"  OR  "evidence based practice"  OR  "evidence-based practice"  OR  outcome ) ) )  AND  ( ( TITLE ( charity  OR  charities  OR  "charitable organisation*"  OR  "voluntary sector"  OR  "voluntary organisations"  OR  "third sector"  OR  third-sector  OR  "community organisation*"  OR  community-based  OR  "community based"  OR  non-profit*  OR  "not for profit"  OR  "nonprofit"  OR  non-profit  "social enterprise*"  OR  tso*  OR  cbo*  OR  vco*  OR  vcs*  OR  "civic sector"  OR  "civil sector"  OR  "social sector"  OR  cso*  OR  "Non Government*"  OR  ngo*  OR  npo*  OR  "civil society" ) ) )

Final Hits: 34
