# Supplementary material for: What are the barriers and facilitators for third sector organisations (non-profits) to evaluate their services? A systematic review
Source: Syst Rev. 2018 Jan 22;7:13. doi: 10.1186/s13643-018-0681-1 (PMC5778760; doi:10.1186/s13643-018-0681-1)
Supplement: Supplementary file 3 — contains the appendices referred to throughout the study. (DOCX 99 kb) [file 13643_2018_681_MOESM1_ESM.docx]

**Appendices for barriers and facilitator review**

**Appendix A:** Adapted checklist for quality appraisal of cross-sectional findings

| **Items** |  | **Yes** | **No** | **Not applicable** | **Unclear** |
| --- | --- | --- | --- | --- | --- |
| *Introduction* | |  |  |  |  |
| 1 | Were the aims/objectives of the study clear? |  |  |  |  |
| *Methods* | |  |  |  |  |
| 2 | Was the study design appropriate for the stated aim(s)? |  |  |  |  |
| 3 | Was the sample size justified? |  |  |  |  |
| 4 | Was the target/reference population clearly defined? (Is it clear who the research was about?) |  |  |  |  |
| 5 | Was the sample frame taken from an appropriate population base so that it closely represented the target/reference population under investigation? |  |  |  |  |
| 6 | Was the selection process likely to select subjects/participants that were representative of the target/reference population under investigation? |  |  |  |  |
| 7 | Were measures undertaken to address and categorise non-responders? |  |  |  |  |
| 8 | Were the risk factor and outcome variables measured appropriate to the aims of the study? (e.g. could you see the questionnaire?) |  |  |  |  |
| 9 | Were the risk factor and outcome variables measured correctly using instruments/measurements that had been trialled, piloted or published previously? |  |  |  |  |
| 10 | Is it clear what was used to determined statistical significance and/or precision estimates? (eg, p values, CIs) |  |  |  |  |
| 11 | Were the methods (including statistical methods) sufficiently described to enable them to be repeated? |  |  |  |  |
| *Results* | |  |  |  |  |
| 12 | Were the basic data adequately described? |  |  |  |  |
| 13 | Does the response rate raise concerns about non-response bias? |  |  |  |  |
| 14 | If appropriate, was information about non-responders described? |  |  |  |  |
| 15 | Were the results internally consistent? |  |  |  |  |
| 16 | Were the results for the analyses described in the methods, presented? |  |  |  |  |
| *Discussion* | |  |  |  |  |
| 17 | Were the authors’ discussions and conclusions justified by the results? |  |  |  |  |
| 18 | Were the limitations of the study discussed? |  |  |  |  |
| *Other* | |  |  |  |  |
| 19 | Were there any funding sources or conflicts of interest that may affect the authors’ interpretation of the results? |  |  |  |  |
| 20 | Was ethical approval or consent of participants attained? |  |  |  |  |

Quality (circle): **High Medium Low**

Reviewer: ________________________________________________

Date: _____________________________________________________________

Study: **__________________________________________________________________________**

Rationale and comment: **____________________________________________________________________________________________________________________________________________________________________________________________________________________________________________________________________________________________________________________________________________________________________________________________________________________________________________________________________________________**

**Appendix B:** Adapted CASP and JBI checklist

| **Checklist questions** | **Yes** | **No** | **Not applicable** | **Unclear** |
| --- | --- | --- | --- | --- |
| Was there a clear statement of the aims |  |  |  |  |
| Is a qualitative methodology appropriate? |  |  |  |  |
| Was the research design appropriate to address the research aims? |  |  |  |  |
| Was the recruitment strategy/sampling appropriate to the aims of the research? |  |  |  |  |
| Was the data collected in a way that addressed the research issue? |  |  |  |  |
| Has the relationship between researcher and participant been adequately considered? |  |  |  |  |
| Have ethical issues been taken into consideration? |  |  |  |  |
| Was the data analysis sufficiently rigorous? |  |  |  |  |
| Is there a clear statement of findings? |  |  |  |  |
| Do the conclusions drawn in the research report flow form the analysis or interpretation of the data? |  |  |  |  |

Quality (circle): **High Medium Low**

Reviewer: ________________________________________________

Date: _____________________________________________________________

Study: **__________________________________________________________________________**

Rationale and comment: **____________________________________________________________________________________________________________________________________________________________________________________________________________________________________________________________________________________________________________________________________________________________________________________________________________________________________________________________________________________**

**Appendix C**: Top 3 cited barriers excluding the six studies of low quality.

| Top 3 cited barriers |
| --- |
| Lack of expertise and internal capacity (14/24) |
| Mismatch between funders requirements and appropriate goals (13/24) |
| Lack of financial resources (10/24) |

**Appendix D**: Themes excluding low quality studies.

| Categories | Times cited |
| --- | --- |
| *Factors associated with technical capacity and evaluation skills* | **35-8=27** |
| Lack of expertise and internal capacity | 17-3=14 |
| Problems with data-collection and analysis | 10-3=7 |
| Inability to utilise existing data | 3-1=2 |
| Difficulty conceptualising and designing evaluation | 1 |
| Technical challenges | 4-1=3 |
| *Factors associated with resources* | **35-9=26** |
| Financial resources | 14-4=10 |
| Staff resources | 6-2=4 |
| Lack of time | 6-1=5 |
| Lack of resources to hire external evaluators | 3-1=2 |
| Resources (unspecified) | 5-1=4 |
| Staff turnover | 1 |
| *Challenges in utilising evaluation systems and identifying outcome indicators* | **25-4=21** |
| Difficulty developing and using evaluation tools | 5 |
| Lack of integrated systems to collect and analyse data | 9-1=8 |
| Challenges in identifying accepted outcome and impact indicators | 12-3=9 |
| *Factors associated with funder requirements* | **23-3=20** |
| Lack of funders requirements and support | 3 |
| Mismatch between funders requirements and appropriate goals | 15-2=13 |
| Poor proportioning of size of charities and funders requirements | 1 |
| Micro-management by donors | 1 |
| Funding insecurity (funding circles incentivising focusing on immediate outputs rather than long terms outcomes) | 3-1=2 |
| *Factors associated with organisational culture and management* | **24-4=20** |
| No perceived benefit to conduct evaluation | 1 |
| Staff resistance to evaluation | 10-1=9 |
| Perceived compromise between evaluation and service delivery | 2 |
| Lack of evaluation strategies and planning | 3-1=2 |
| Lack of feedback between board and management staff | 1 |
| Prioritisation | 2-1=1 |
| Lack of support from board and leadership | 4-1=3 |
| Evaluation not part of everyday practice | 1 |
| *Other* | **5-1=4** |
| Confidentiality of data | 3-1=2 |
| Lack of cooperation with stakeholders | 2 |
| Factors operating as facilitators |  |
| *Factors related to receiving support to evaluate* | **19-1=18** |
| Partnering with evaluation experts | 3 |
| Partnering with organisations working with similar activities | 1 |
| Technology availability and literacy to collect and analyse data | 4 |
| Benchmark data availability | 2 |
| Training of staff and evaluation literacy | 5-1 |
| Workable evaluation tools | 2 |
| Having sufficient resources to evaluate | 2 |
| *Factors related to organisational culture and management* | **18-1=17** |
| Understanding internal processes | 3-1 |
| To embed evaluation as part of everyday practice | 1 |
| Improve allocation of resources | 1 |
| Support from board and leadership | 4 |
| Have in-house evaluation staff | 3 |
| Having clear goals and evaluation strategies | 2 |
| Staff support | 3 |
| Positive perception of evaluation |  |
| *Factors related to the motivation to be accountable* | **17** |
| Involvement of stakeholders to identify outcome indicators and evaluation goals | 5 |
| The motivation to influence policy | 1 |
| Compare work and outcomes to others doing similar work | 1 |
| The motivation to inform the sector as whole | 1 |
| Improve targeting of beneficiaries | 1 |
| Identify new approaches | 1 |
| Ensuring control and legitimacy of activities to stakeholders | 3 |
| The motivation to demonstrate and improve effectiveness of services | 4 |
| *Factors around funder requirements and regulations* | **5** |
| Funder requirements | 4 |
| Regulation requirements | 1 |
| *Factors around economic sustainability* | **5** |
| Using evaluation to be eligible for funding opportunities | 3 |
| Using evaluation as marketing | 2 |
